# Verification of a method using magnetic bead enrichment and nucleic acid extraction to improve the molecular detection of bacterial contamination in blood components

**DOI:** 10.1128/spectrum.02760-23

**Published:** 2024-02-06

**Authors:** Byungjoon Na, Jinyeop Lee, Ho Eun Chang, Eunseon Park, Sojin Park, Jiyoung Lee, Sujin Oh, Dong Woo Shin, Yun Ji Hong, Kyoung Un Park

**Affiliations:** 1 KingoBio Inc. Research Center, Seoul, South Korea; 2 PHiCS Institute, Seoul, South Korea; 3 Department of Laboratory Medicine, Seoul National University College of Medicine, Seoul, South Korea; 4 Department of Laboratory Medicine, Seoul National University Bundang Hospital, Seongnam, South Korea; Michigan State University, East Lansing, Michigan, USA

**Keywords:** bacterial contamination, platelets, magnetic nanobeads, real-time PCR, bacterial enrichment

## Abstract

Bacterial contamination of blood products poses a significant risk in transfusion medicine. Platelets are particularly vulnerable to bacterial growth because they must be stored at room temperature with constant agitation for >5 days. The limitations of bacterial detection using conventional methods, such as blood cultures and lateral flow assays, include the long detection times, low sensitivity, and the requirement for substantial volumes of blood components. To address these limitations, we assessed the performance of a bacterial enrichment technique using antibiotic-conjugated magnetic nanobeads (AcMNBs) and real-time PCR for the detection of bacterial contamination in plasma. AcMNBs successfully captured >80% of four bacterial strains, including *Staphylococcus aureus*, *Bacillus cereus*, *Escherichia coli*, and *Klebsiella pneumoniae*, in both plasma and phosphate-buffered saline. After 24-h incubation with bacterial enrichment, *S. aureus* and *B. cereus* were each detected at 10^1^ CFU/mL in all trials (5/5), *E. coli* at 10^1^ CFU/mL in 1/5 trials, and *K. pneumoniae* at 10² CFU/mL in 4/5 trials. Additionally, without incubation, the improvement was also achieved in samples with bacterial enrichment, *S. aureus* at 10² CFU/mL and *B. cereus* at 10^1^ CFU/mL in 1/5 trials each, *E. coli* at 10³ CFU/mL in 3/5 trials, and *K. pneumoniae* at 10¹ CFU/mL in 2/5 trials. Overall, the findings from this study strongly support the superiority of bacterial enrichment in detecting low-level bacterial contamination in plasma when employing AcMNBs and PCR.

**IMPORTANCE:**

The study presents a breakthrough approach to detect bacterial contamination in plasma, a critical concern in transfusion medicine. Traditional methods, such as blood cultures and lateral flow assays, are hampered by slow detection times, low sensitivity, and the need for large blood sample volumes. Our research introduces a novel technique using antibiotic-conjugated magnetic nanobeads combined with real-time PCR, enhancing the detection of bacteria in blood products, especially platelets. This method has shown exceptional efficiency in identifying even low levels of four different species of bacteria in plasma. The ability to detect bacterial contamination rapidly and accurately is vital for ensuring the safety of blood transfusions and can significantly reduce the risk of infections transmitted through blood products. This advancement is a pivotal step in improving patient outcomes and elevating the standards of care in transfusion medicine.

## INTRODUCTION

Bacterial contamination of platelets is a major problem in blood management and transfusion medicine (1, 2). Platelets are at particularly high risk for bacterial contamination because they must be stored at room temperature with agitation, typically for up to 5 days (3–5). Under these conditions, bacteria can easily proliferate from 1 CFU/mL to >10^8^ CFU/mL (6). The transfusion of contaminated platelets to a patient can result in an immediate transfusion-transmitted bacterial reaction, including high fever, nausea, and hypotension, which can be fatal (7, 8). According to a recent study, the estimated rate of bacterial contamination per transfused unit on the day of transfusion is 1/2,500 (9), and fatal septic shock due to the transfusion of a contaminated platelet concentrate is not uncommon (10, 11). The Food and Drug Administration (FDA) reported 21 deaths due to the transfusion of contaminated platelets from 2011 to 2020 (12). Thus, while the number of cases is small, morbidity and mortality associated with these incidents are very high (13, 14).

The bacteria most often associated with infections related to platelet contamination are *Staphylococcus aureus*, *Escherichia coli*, *Bacillus cereus*, and *Klebsiella pneumoniae* (15–17). The gold standard method for detecting such contamination is blood culture, typically using the BacT/Alert system (Organon Teknika Corp., Durham, NC, USA) (18–20). However, this has two major limitations: it takes 24 h to several days to detect bacteria and it requires 5–10 mL of blood (21, 22). In addition, automated blood culture systems may yield false-negative results due to low bacterial titers and slow bacterial growth (23). Association for the Advancement of Blood & Biotherapies and the FDA have established specific methodologies and guidelines for preventing, detecting, and monitoring the bacterial contamination of blood products, especially platelets (24, 25), in addition to highly recommending product sterilization to prevent contamination (26, 27). For example, bacterial pathogens in blood products can be inactivated by low pH incubation and nano-filtration (28, 29), but their efficacy and safety for use in blood transfusion remain to be established (30). A rapid and precise diagnostic method for detecting bacterial contamination in platelets is therefore needed.

Among the efforts to develop rapid, sensitive methods for the detection of bacterial pathogens in blood products (31–34) is the enhanced bacterial detection system (eBDS; Haemonetics Corp., Braintree, MA, USA), which identifies bacteria by measuring changes in the oxygen concentration over 24 h, but it does not allow the detection of anaerobic bacteria (35). The platelet pan-genera detection (PGD) test (Verax Biomedical, Marlborough, MA, USA) is a 30-min lateral flow immunoassay that detects lipoteichoic acid in Gram-positive bacteria and lipopolysaccharide in Gram-negative bacteria (36). However, the sensitivity of the test is low (~10^4^ CFU/mL) and the false-positive rate is high (37). Nucleic acid amplification using real-time PCR is a highly sensitive and specific method for detecting bacterial pathogens (38, 39), but molecular systems that can verify the safety of blood components for transfusions have yet to be developed and molecular diagnosis is not a feature of either the PGD test or the eBDS. Moreover, platelets contain IgG and other substances that can interfere with nucleic acid amplification (40). Thus, the success of a molecular system requires the separation of bacterial DNA from these inhibitors.

In previous studies, we developed a method in which magnetic beads immobilized with antibiotics were used to enrich spiked samples of Gram-negative and Gram-positive bacteria in apheresis plasma (41). Then the bacterial DNA was extracted and detected by real-time PCR. Extending this approach, our current study assessed its effectiveness against a broader spectrum of bacteria, including two Gram-negative (*E. coli* and *K. pneumoniae*) and two Gram-positive (*S. aureus* and *B. cereus*) bacteria. The primary aim is to efficiently isolate bacteria from 500 µL of apheresis plasma using our sensitive and precise enrichment process. We recognize that using larger sample volumes could more effectively show our method’s capabilities. However, practical constraints in transfusion medicine, such as the limited availability of plasma in clinical settings, made it essential to work with optimal volumes.

## MATERIALS AND METHODS

### Bacterial strains

*S. aureus*, *B. cereus*, *E. coli*, and *K. pneumoniae* were cultured overnight at 37°C, 150 rpm, in 5 mL of Luria-Bertani (LB) broth (Sigma-Aldrich, St. Louis, MO, USA). Then 50 µL cultured samples was inoculated into 5 mL of LB broth and cultured for 3 h under the same conditions. When the optical density at 600 nm (OD_600_) reached 1, the concentration of *B. cereus* was determined to be 10^7^ CFU/mL, and the concentration of the other three bacterial strains was found to be 10^8^ CFU/mL (42–45). Bacterial pellets were harvested by centrifuging 1 mL cultured sample at 8,000 rpm and room temperature for 10 min. The cells in the pellets were suspended in 1 mL of phosphate-buffered saline (PBS; Gibco, Grand Island, NY, USA).

### Preparation of antibiotic-conjugated magnetic nanobeads

**Fig 1:**
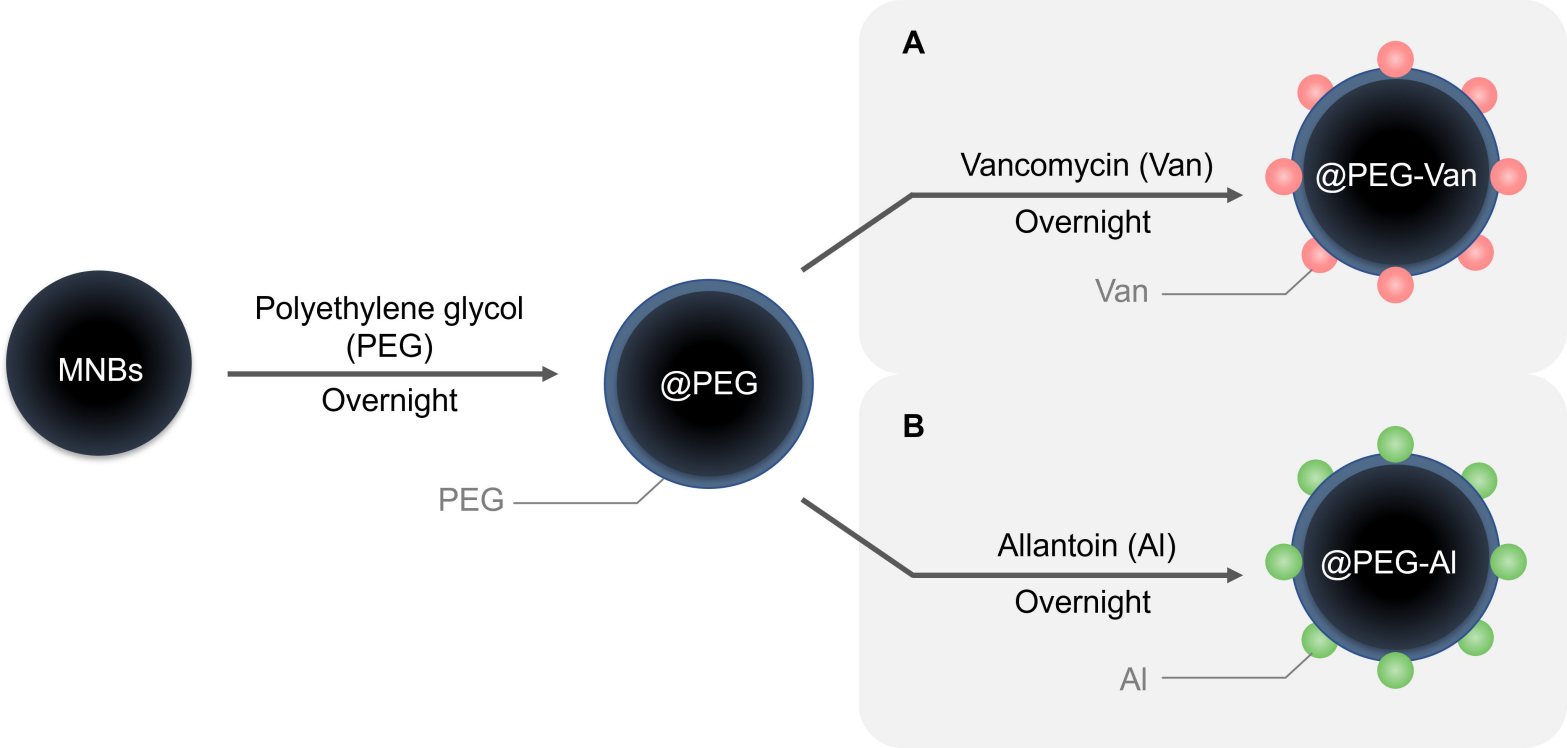
Schematic diagram of the protocol for conjugating antibiotics with magnetic nanobeads. (**A**) Synthesis scheme of MNBs@PEG-Van and (**B**) MNBs@PEG-Al.

In this work, the magnetization of magnetic nanobeads (MNBs), which form the core of the antibiotic-conjugated magnetic nanobeads (AcMNBs), was improved compared to those used in our previous studies (Fig. S1). Magnetization was increased by exposing the beads to a solution in which the FeCl_3_ . 6H_2_O/sodium acetate anhydrous (Fe/NaOAc; Sigma-Aldrich) molar ratio was ≤0.8 and by reducing the synthesis time according to the Fe/NaOAc molar ratio (46). The subsequent coating process was the same as described previously (41). Briefly, 200 mg of MNBs was dispersed in 40 mL of 1 M HCl (Sigma-Aldrich), stirred at room temperature for 1 h, separated from the HCl by washing with PBS using a magnetic rack (Bioneer Corp., Daejeon, South Korea), and dispersed in 10 mL of PBS. Then 50 mg of MNBs was mixed overnight at room temperature with 25 mg of polyethylene glycol (PEG; Sigma-Aldrich) and 25 mL of Tris-buffer (Bioneer Corp.). Vancomycin conjugates of the MNBs@PEG-COOH (Van-MNBs@PEG-COOH) were obtained by stirring a suspension of MNBs@PEG-COOH, prepared by adding 5 mg of MNBs@PEG-COOH to 500 µL of MES buffer, together with the Van-EDC-NHS suspension at room temperature for 2 h. The Van-EDC-NHS suspension was prepared by adding 7 mg of N-hydroxysuccinimide (NHS), 4 mg of 1-ethyl-3-(3-dimethylaminopropyl)carbodiimide (EDC), and 10 mg of Van to 1 mL of 2-(N-morpholino)ethanesulfonic acid (MES) buffer. The MNBs@polyethylene glycol-vancomycin (MNBs@PEG-Van) was washed with PBS and suspended in PBS at 4°C. MNBs@polyethylene glycol-allantoin (MNBs@PEG-Al) was prepared in the same way except that Van was replaced with allantoin (Al; Sigma-Aldrich). Figure 1 shows the overall process of bead preparation.

### Preparation for bacteria-spiked plasma

The bacterial suspensions were serially diluted with PBS to a final concentration of 10^5^ CFU/mL and used to spike 500 µL of plasma to achieve concentrations of 10^1^ to 10^4^ CFU/mL by serial dilution. Plasma was prepared from residual apheresis plasma as a blood component for transfusion. The bacteria-spiked plasma was incubated or not for 24 h and then exposed to the AcMNBs for enrichment or not as described below. Our study received an exemption from Institutional Review Board review, designated with the number X-2109-709-901. The apheresis plasma used in our research was obtained from residual apheresis plasma following transfusion procedures, ensuring ethical compliance and practical relevance to the pilot test.

### Bacterial enrichment by AcMNBs in plasma

Bacteria-spiked plasma samples were prepared with and without 24-h incubation. MNBs@PEG-Van was used to enrich Gram-positive bacteria, and MNBs@PEG-Al was used to enrich Gram-negative bacteria. In both, 0.4 mg of AcMNBs was mixed with 500 µL of bacteria-spiked plasma samples and incubated on a rotary platform at room temperature for 20 min. A magnetic rack was used to separate the bacteria–AcMNB complexes from the supernatant plasma. The complexes were washed twice with 1 mL of PBS and dispersed in 200 µL of PBS.

### Bacterial capture efficiency by AcMNBs in plasma

To assess the bacterial capture efficiency of AcMNBs in plasma, we commenced by preparing four bacterial species at a concentration of 10^4^ CFU/mL in bacteria-spiked plasma. Then, 0.4 mg of AcMNBs was added to 500 µL of this spiked plasma. This mixture was then allowed to react at room temperature for 20 min. Following the reaction, bacteria–AcMNB clusters were separated using a magnetic rack. The resulting supernatant were diluted at a 1:10 ratio. Subsequently, 50 µL aliquots of these dilutions were plated onto LB agar plates, which were then incubated at 37°C for 16 h. To ensure consistency and minimize variability, the mean and standard deviation (SD) of the capture efficiencies were determined based on an analysis of five separate plates.

Bacterial capture efficiency was calculated using the following formula: capture efficiency (%) = (*N_a_* − *N_b_*/*N_a_*) × 100, where “*N*_*a*_” denotes the bacterial concentration in the sample before enrichment and “*N*_*b*_” represents the concentration remaining in the supernatant post-enrichment. The mean and SD of these capture efficiencies were derived from the data obtained from five plates.

### Field emission scanning electron microscopy sample preparation of bacteria binding to AcMNBs

The morphology of *S. aureus*, *B. cereus*, *E. coli*, and *K. pneumoniae* after capture by AcMNBs was evaluated using field emission scanning electron microscopy (FE-SEM; JSM-7800F Prime, JEOL Ltd., Tokyo, Japan). The FE-SEM samples were prepared as follows: bacteria–AcMNB complexes were fixed in 2% (wt/vol) glutaraldehyde (Sigma-Aldrich) at room temperature for 1 h, washed three times with 1 mL of PBS, dehydrated in an ethanol (30%, 50%, 70%, 80%, 90%, and 100%) series for 10 min/step, and incubated for 1 h in 1% (wt/vol) osmium tetroxide (Sigma-Aldrich) at 4°C in the dark. Then 20 µL of each bacteria–AcMNB complex suspension was placed dropwise onto carbon-coated copper grids (Electron Microscopy Sciences, Hatfield, PA, USA), dried at room temperature, and observed using a JSM-7800F Prime microscope.

### Bacterial DNA extraction from bacteria-spiked plasma with and without incubation

**Fig 2:**
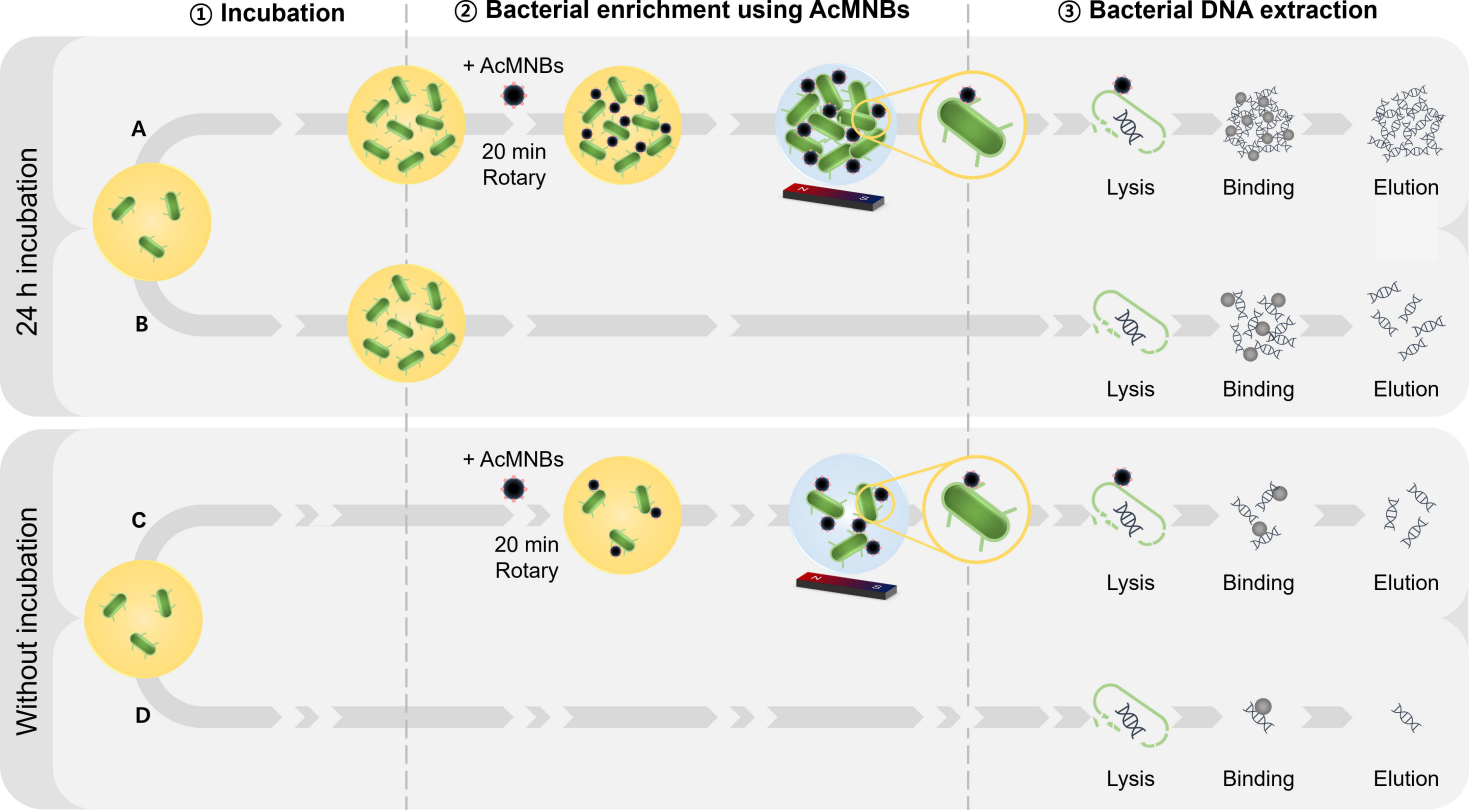
Schematic diagram of the methodology used in this study. (**A**) Bacterial DNA extraction with 24-h incubation and bacterial enrichment. (**B**) Bacterial DNA extraction with 24-h incubation. (**C**) Bacterial DNA extraction without incubation and with bacterial enrichment. (**D**) Bacterial DNA extraction without incubation.

The bacterial samples were incubated or not for 24 h and then enriched or not as described below (Fig. 2). Bacterial DNA was extracted from all bacteria–AcMNB complexes using the K-SL DNA extraction kit (KingoBio Inc., Seoul, South Korea). The extracted DNA was stored at –80°C. In addition, plasma spiked with Gram-positive bacteria was prepared by adding 10 µL of lysostaphin (Sigma-Aldrich) to each sample before the lysis step, followed by a 10-min incubation at room temperature.

### Verification of specificity of AcMNBs through molecular detection of extracted DNA in bacteria-free plasma of blood components

This study involved a total of 60 residual apheresis plasma samples, each consisting of 500 µL. The samples were divided into two groups: one group of 30 samples was incubated for 24 h, while the other group of 30 samples was not incubated. All samples were initially confirmed to be free of bacteria. For the enrichment process, two types of AcMNBs were used: MNBs@PEG-Van and MNBs@PEG-AI. In each group, 15 samples from the 24-h incubated set and 15 non-incubated samples were treated with 0.4 mg of MNBs@PEG-Van. Similarly, another set of 15 incubated samples and 15 non-incubated samples was treated with 0.4 mg of MNBs@PEG-AI. The samples were then incubated on a rotary platform at room temperature for 20 min. A magnetic rack was used to separate the AcMNBs from the supernatant plasma. The samples with MNBs@PEG-Van were used for the extraction of DNA from Gram-positive bacteria, while those with MNBs@PEG-AI were used for the extraction of DNA from Gram-negative bacteria. For the samples with MNBs@PEG-Van, we used primers for Gram-positive bacteria to amplify and observe the results. Similarly, for the samples with MNBs@PEG-AI, we employed primers for Gram-negative bacteria for amplification.

### Real-time PCR assay

The enriched samples were extracted to an elution volume of 50 µL from 500 µL, and the non-enriched samples were extracted to an elution volume of 50 µL from 200 µL using the K-SL protocol in all extractions, regardless of incubation. Out of the 50 µL elution volume, only 2 µL was used for real-time PCR. The extracted bacterial DNA was analyzed via real-time PCR and the bacterial species were subsequently identified. The primers were designed using PrimerQuest (Integrated DNA Technologies Inc., Coralville, IA, USA); the primer sequences are listed in Table 1. The samples were prepared in Power SYBR Green PCR Master Mix (Applied Biosystems, Waltham, MA, USA). The 25 µL reaction mix contained 2.5 µL sample DNA, 12.5 µL Power SYBR Green PCR Master Mix, 9 µL distilled H_2_O, and 5 pmol of each specific primer (Table 1). Amplification consisted of 40 cycles of 15 s at 95°C, 15 s at 58°C, and 30 s at 72°C. Real-time PCR was performed using the QuantStudio 3 system (Applied Biosystems). Real-time PCR using bacteria-spiked plasma was repeated five times to calculate the positivity rate and the mean and SD of the threshold cycle (Ct). Positive controls contained DNA directly extracted from cultured colonies of *S. aureus*, *B. cereus*, *E. coli*, and *K. pneumoniae*. The negative control was RNase-free and DNase-free water. A result was considered positive when the Ct was <38.0, determined using the appropriate specific melting temperature: 76.5 ± 0.5°C for *S. aureus*, 79.0 ± 0.5°C for *B. cereus*, 84.5 ± 0.5°C for *E. coli*, and 83.5 ± 0.5°C for *K. pneumoniae*.

**TABLE 1:** Primers used in the real-time PCR assay

Target bacteria	Target gene	Primer	Sequences (5′−3′)	Length (bp)	Reference
*S*. *aureus*	nuc	nuc_F nuc_R	TATGGACGTGGCTTAGCGTAT GACCTGAATCAGCGTTGTCTT	193	This study
*B*. *cereus*	DUF3817	Bcg_F Bcg_R	AACAGGCTCCATACAATCCTAT TGGTAGCGTTTCTTCGTCTTAT	250	(47)
*E*. *coli*	tyrB	tyB_F tyB_R	AAGAGGATGCCTACGCCATT CTTGGCGGGCTGGAGTAGTT	199	This study
*K*. *pneumoniae*	tyrB	tyrB_F tyrB_R	CCTCGCTGTATCTGCCAATG ACCGTCTCAATAGAGGCAATG	118	This study

## RESULTS

**Fig 3:**
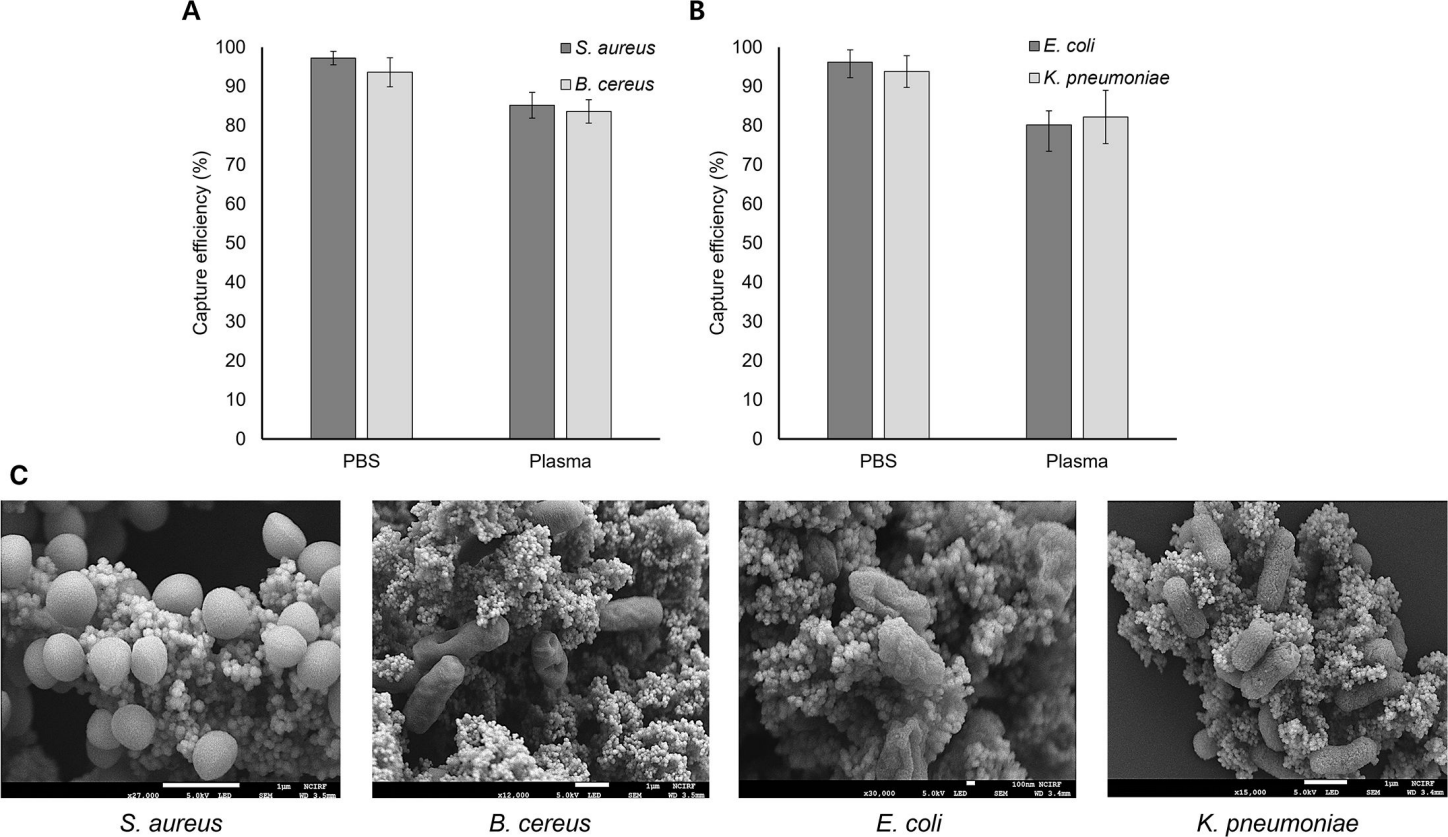
Capture efficiencies of four species of bacteria using AcMNBs and the FE-SEM images of the bacteria–AcMNB complexes. (**A**) Sample type-dependent capture efficiencies of Gram-positive bacteria (*S. aureus* and *B. cereus*) using MNBs@PEG-Van. (**B**) Sample type-dependent capture efficiencies of Gram-negative bacteria (*E. coli* and *K. pneumoniae*) using MNBs@PEG-Al. (**C**) FE-SEM images of Gram-positive bacteria (*S. aureus* and *B. cereus*) captured by MNBs@PEG-Van and Gram-negative bacteria (*E. coli* and *K. pneumoniae*) captured by MNBs@PEG-Al.

The capture efficiencies of the four species of bacteria are shown in Fig. 3 together with the FE-SEM images of the AcMNB-captured bacteria. Gram-positive bacteria (*S. aureus* and *B. cereus*) were captured in PBS and plasma using MNBs@PEG-Van. The capture efficiency in PBS was 97.2 ± 1.7% for *S. aureus* and 93.6 ± 3.7% for *B. cereus*, and in plasma, it was 85.2 ± 3.3% and 83.6 ± 3.0%, respectively (Fig. 3A). Gram-negative bacteria (*E. coli* and *K. pneumoniae*) were captured in PBS and plasma using MNBs@PEG-Al. The capture efficiency in PBS was 96.2 ± 3.1% for *E. coli* and 93.8 ± 4.0% for *K. pneumoniae* and that in plasma was 80.2 ± 3.6% and 82.2 ± 6.8%, respectively (Fig. 3B). The FE-SEM images of Gram-positive bacteria (*S. aureus* and *B. cereus*) captured by MNBs@PEG-Van and Gram-negative bacteria (*E. coli* and *K. pneumoniae*) captured by MNBs@PEG-Al showed that the AcMNBs effectively captured the bacteria (Fig. 3C).

### Real-time PCR in bacteria-spiked plasma with and without bacterial enrichment

**Fig 4:**
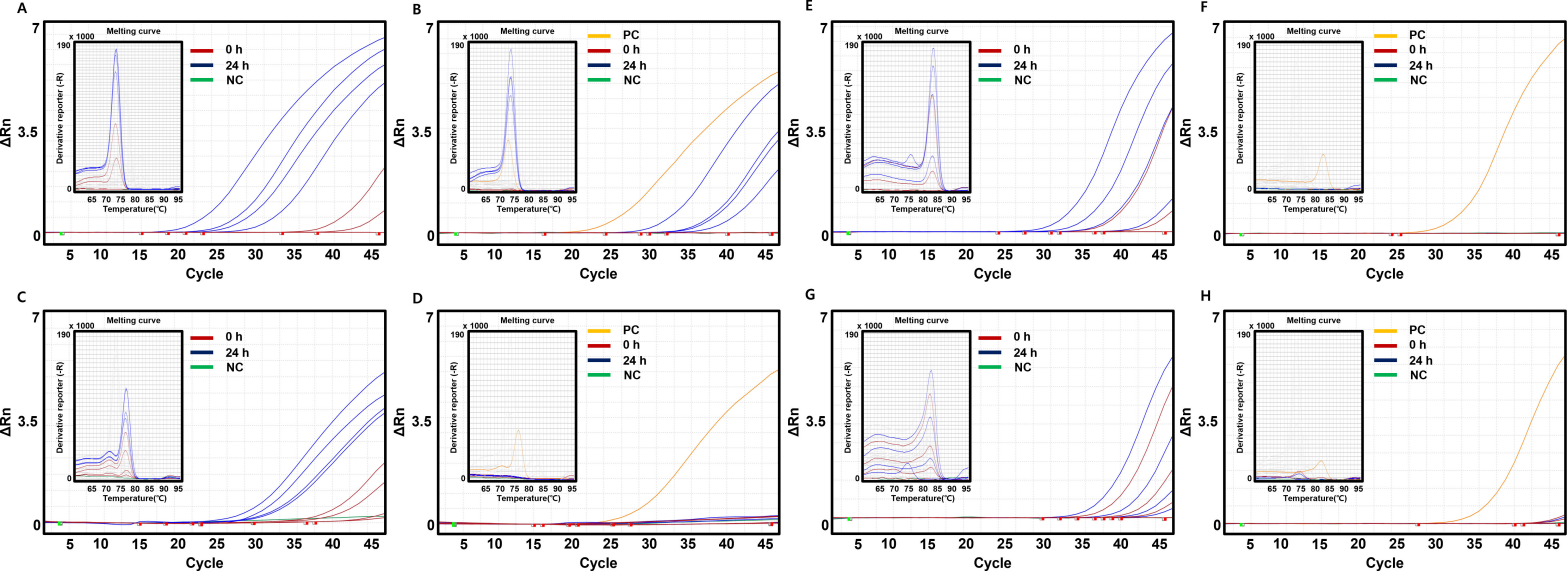
Results of real-time PCR in bacteria-spiked plasma with and without bacterial enrichment. Amplification and melting curves are shown. Each concentration range (10^1^ to 10^4^ CFU/mL) is represented by a uniform color scheme. Red lines indicate samples that were not incubated (0 h), while purple lines represent samples incubated for 24 h. The positive control (PC) is denoted by yellow lines, and the negative control (NC) is illustrated with green lines. (**A**) *S. aureus*-specific PCR results with bacterial enrichment, (**B**) *S. aureus*-specific PCR results without bacterial enrichment, (**C**) *B. cereus*-specific PCR results with bacterial enrichment, (**D**) *B. cereus*-specific PCR results without bacterial enrichment, (**E**) *E. coli*-specific PCR results with bacterial enrichment, (**F**) *E. coli*-specific PCR results without bacterial enrichment, (**G**) *K. pneumoniae*-specific PCR results with bacterial enrichment, and (**H**) *K. pneumoniae*-specific PCR results without bacterial enrichment.

For 1 out of 5 positivity rates, the SD could not be calculated; for 0 out of 5 positivity rates, the mean and SD could not be calculated. Positivity rates of bacterial DNA extraction in samples incubated or not for 24 h were higher with enrichment than without enrichment. Fig. 4A demonstrates that when bacterial enrichment was applied after 24-h incubation, *S. aureus* was detected at 10 CFU/mL in all five repetitions (5/5). However, without incubation, detection was possible at 10^2^ CFU/mL in 1/5 trials and at 10^3^ and 10^4^ CFU/mL in 3/5 trials. Fig. 4B shows the results for *S. aureus* without bacterial enrichment, where after 24-h incubation, 10 CFU/mL was detected in all five repetitions (5/5). Without incubation, 10^4^ CFU/mL of *S. aureus* was detected in 2/5 trials. Fig. 4C indicates that with bacterial enrichment after 24-h incubation, *B. cereus* was detected at 10 CFU/mL in all five repetitions (5/5). Without incubation, detection was possible at 10 CFU/mL in 1/5 trials and at 10^2^ CFU/mL in 2/5 trials. Fig. 4D shows the results for *B. cereus* without bacterial enrichment, where after 24-h incubation, 10^3^ CFU/mL of *B. cereus* was detected in 3/5 trials at 10^4^ CFU/mL in 4/5 trials. However, without incubation, 10^4^ CFU/mL of *B. cereus* was detected in 1/5 trials.

In Fig. 4E, with bacterial enrichment after 24-h incubation, *E. coli* was detected at 10 CFU/mL in 1/5 trials. Additionally, without incubation, *E. coli* was detected at 10 CFU/mL in 1/5 trial and at 10^3^ CFU/mL in 3/5 trials. Fig. 4F shows the results for *E. coli* without bacterial enrichment, where after 24-h incubation, 10^3^ CFU/mL was detected in only 1/5 trials. Without incubation, 10^3^ CFU/mL of *E. coli* was detected in 2/5 trials. Fig. 4G reveals that with bacterial enrichment and 24-h incubation, *K. pneumoniae* was detected at 10^2^ CFU/mL in 4/5 trials. Without incubation, *K. pneumoniae* was detected at 10 CFU/mL in 2/5 trials and at 10^2^ CFU/mL in 5/5 trials. Fig. 4H shows the results for *K. pneumoniae* without bacterial enrichment, where after 24-h incubation, 10^4^ CFU/mL was detected in 2/5 trials. However, without incubation, 10^4^ CFU/mL of *K. pneumoniae* was detected in 1/5 trials. The detailed numerical values of Fig. 4 are presented in Table 2.

**TABLE 2:** Results of real-time PCR analysis in bacteria-spiked plasma

Bacteria	Concn (CFU^*a*^/mL)	Without incubation							24-h incubation						
		Without bacterial enrichment			With bacterial enrichment			Mean Ct difference^*a*^	Without bacterial enrichment			With bacterial enrichment			Mean Ct difference
		Positive rate	Ct		Positive rate	Ct			Positive rate	Ct		Positive rate	Ct		
			Mean	SD		Mean	SD			Mean	SD		Mean	SD	
*S. aureus*	10^4^	2/5	36.62	0.68	3/5	34.79	1.15	1.83	5/5	25.45	3.57	5/5	18.17	0.95	7.28
	10^3^	0/5	NA^*b*^	NA	3/5	35.33	2.15	NA	5/5	27.20	3.75	5/5	20.04	1.21	7.16
	10^2^	0/5	NA	NA	1/5	37.42	NA	NA	4/5	27.06	5.73	5/5	22.65	1.21	4.42
	10^1^	0/5	NA	NA	0/5	NA	NA	NA	5/5	29.24	4.59	5/5	27.35	1.25	1.89
*B. cereus*	10^4^	1/5	27.71	NA	5/5	31.42	2.48	−3.72	4/5	29.48	3.34	5/5	21.94	1.11	7.53
	10^3^	0/5	NA	NA	5/5	34.26	2.10	NA	3/5	30.73	5.06	5/5	23.34	1.85	7.39
	10^2^	0/5	NA	NA	2/5	34.08	3.44	NA	0/5	NA	NA	5/5	24.27	2.01	NA
	10^1^	0/5	NA	NA	1/5	37.36	NA	NA	0/5	NA	NA	5/5	24.72	1.04	NA
*E. coli*	10^4^	0/5	NA	NA	5/5	33.15	1.03	NA	2/5	34.89	2.15	5/5	28.41	1.91	6.48
	10^3^	2/5	36.79	1.56	3/5	36.22	1.73	0.56	1/5	33.25	NA	5/5	31.88	1.26	1.38
	10^2^	0/5	NA	NA	0/5	NA	NA	NA	0/5	NA	NA	5/5	34.91	0.59	NA
	10^1^	0/5	NA	NA	0/5	NA	NA	NA	0/5	NA	NA	1/5	36.53	NA	NA
*K. pneumoniae*	10^4^	1/5	37.12	NA	5/5	29.63	1.34	7.49	2/5	33.40	2.06	5/5	26.78	1.20	6.62
	10^3^	0/5	NA	NA	4/5	32.70	0.54	NA	0/5	NA	NA	5/5	30.78	1.61	NA
	10^2^	0/5	NA	NA	5/5	34.26	1.09	NA	0/5	NA	NA	4/5	34.16	0.91	NA
	10^1^	0/5	NA	NA	2/5	34.18	0.21	NA	0/5	NA	NA	0/5	NA	NA	NA

^*a*^ Mean Ct difference, difference of Ct mean between with bacterial enrichment and without bacterial enrichment.

^*b*^ NA, not applicable due to the result of not detected or lack of data.

### Verification of specificity of AcMNBs through real-time PCR of extracted DNA in bacteria-free plasma of blood components

**Fig 5:**
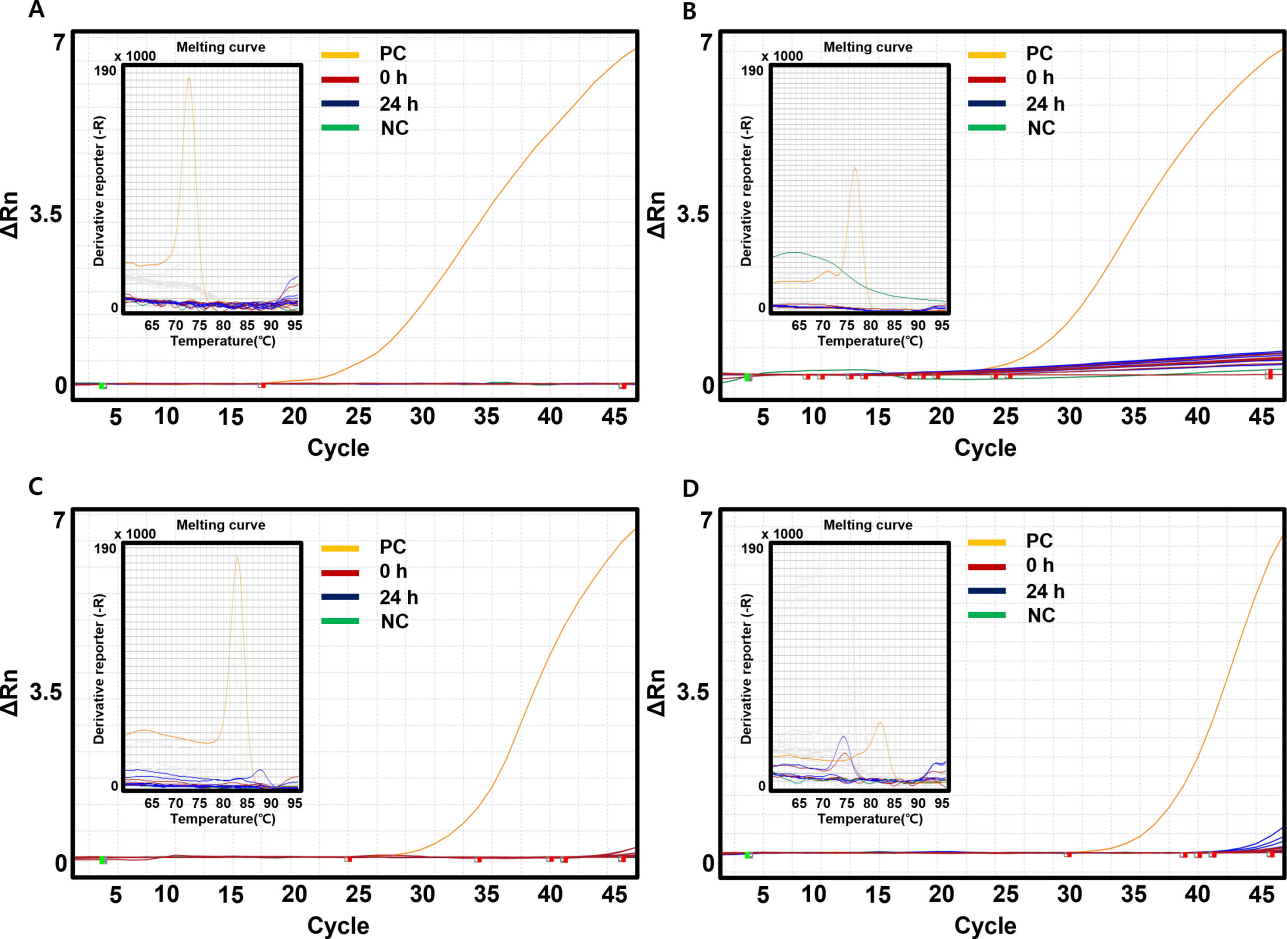
Results of real-time PCR in bacteria-free plasma of blood components with enrichment by AcMNBs. Amplification and melting curves are shown. Each concentration range (10^1^ to 10^4^ CFU/mL) is represented by a uniform color scheme. Red lines indicate samples that were not incubated (0 h), while purple lines represent samples incubated for 24 h. The positive control (PC) is denoted by yellow lines, and the negative control (NC) is illustrated with green lines. (**A**) *S. aureus*-specific PCR results. (**B**) *B. cereus*-specific PCR results. (**C**) *E. coli*-specific PCR results. (**D**) *K. pneumoniae*-specific PCR results.

All results from the real-time PCR analysis conducted on bacteria-free plasma of blood components, enriched with AcMNBs, indicated no detection of bacterial DNA (Fig. 5). Across all 60 samples, both incubated and without incubated, the real-time PCR test for the targeted species (*S. aureus*, *B. cereus*, *E. coli*, and *K. pneumoniae*) yielded negative results, corroborating the specificity of AcMNBs in excluding non-target DNA. This finding is clearly illustrated in Fig. 5.

## DISCUSSION

The effectiveness of the magnetic bead-based enrichment and nucleic acid extraction method in enhancing the performance of molecular detection of bacterial contamination in plasma was tested in 380 samples, comprising 320 bacteria-spiked samples and 60 bacteria-free plasma samples. The tested bacteria consisted of two species of Gram-positive bacteria and two species of Gram-negative bacteria. The efficacy of the AcMNBs was verified using the K-SL DNA extraction kit. In the case of the enriched samples, we began with 500 µL of each sample, followed by an enrichment process. After enrichment, the supernatant was discarded, and the sample was resuspended in 200 µL of buffer for DNA extraction. Thus, the starting volume for DNA extraction remained consistent across both enriched and unenriched samples. The specificity of the AcMNBs was high as there were no false positives in the bacteria-free samples.

To achieve broad bacterial capture, Van was used to capture Gram-positive bacteria, and Al was used to capture Gram-negative bacteria. Van is a glycopeptide antibiotic that binds via the D-Ala-D-Ala end of peptidoglycan to inhibit cell wall biosynthesis in Gram-positive bacteria (48). Al selectively binds to the lipopolysaccharides that constitute a major fraction of the outer membrane of Gram-negative bacteria (49). Both MNBs@PEG-Van and MNBs@PEG-Al showed high capture efficiencies (>90%) when tested in PBS for all species of Gram-positive and Gram-negative bacteria, respectively. However, the capture efficiency in plasma was slightly lower (80%). The difference can be attributed to the presence of various proteins in plasma that inhibit binding between bacteria and beads, including fibrinogen, albumin, and globulin (50). These proteins adhere to AcMNBs, resulting in aggregates that prevent contact between the bead surface and bacteria. Unspecific binding can be reduced by coating the beads with PEG (51).

Regardless of the presence or absence of incubation, the bacterial enrichment process based on AcMNBs significantly enhanced the detection rates of bacteria in spiked samples by at least 10 to 100 times compared to samples without enrichment. The AcMNBs exhibited excellent capture efficiencies and specificity for both Gram-positive and Gram-negative bacteria. This efficiency is attributed to the effective separation and enrichment of bacteria from plasma, facilitating the acquisition of more abundant and purified bacterial DNA. These results demonstrate that bacterial contamination in plasma can be efficiently detected after 24 h of incubation followed by bacterial enrichment and DNA extraction. Therefore, our study highlights the potential of AcMNBs coupled with PCR for the capture and detection of bacterial pathogens in plasma samples.

The ability of AcMNBs to efficiently capture and enrich bacterial pathogens from plasma combined with the specificity and sensitivity of PCR detection suggests that this method could be used in an automated diagnostic system. Moreover, by reducing the need for extensive sample preparation and manual handling, this method could potentially reduce the time and cost of diagnosing bacterial infections. Future study endeavors should focus on the development of AcMNBs that are adept at simultaneously capturing both Gram-positive and Gram-negative bacteria. Additionally, efforts should be directed toward reducing the incubation time required in these processes. We plan to optimize the use of AcMNBs and PCR in automated diagnostic platforms and evaluate the performance of this approach in larger sample sizes and with a wider range of bacterial species.

## SUPPLEMENTAL MATERIAL

Figure S1 spectrum.02760-23-s0001.pdf Comparison of particle collection time.

ASM does not own the copyrights to Supplemental Material that may be linked to, or accessed through, an article. The authors have granted ASM a non-exclusive, world-wide license to publish the Supplemental Material files. Please contact the corresponding author directly for reuse.
