# Supplementary material for: Verification of a method using magnetic bead enrichment and nucleic acid extraction to improve the molecular detection of bacterial contamination in blood components
Source: Microbiol Spectr. 2024 Feb 6;12(3):e02760-23. doi: 10.1128/spectrum.02760-23 (PMC10913752; doi:10.1128/spectrum.02760-23)
Supplement: Figure S1 — Comparison of particle collection time. [file spectrum.02760-23-s0001.pdf]

Supplementary Materials

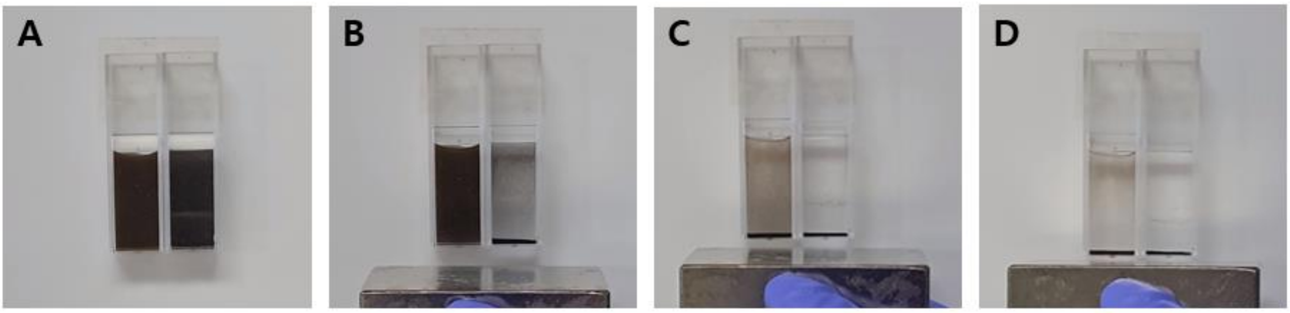

Supplementary Figure 1.

Comparison of particle collection time between the previous MNBs (left) and the MNBs used in this study (right)

A. Before attaching the magnet

B. After attaching the magnet for 1 sec

C. After attaching the magnet for 5 sec

D. After attaching the magnet for 10 sec
